# Autophagy induces apoptosis and death of T lymphocytes in the spleen of pigs infected with CSFV

**DOI:** 10.1038/s41598-017-14082-9

**Published:** 2017-10-19

**Authors:** Hongchao Gou, Mingqiu Zhao, Shuangqi Fan, Jin Yuan, Jiedan Liao, Wencheng He, Hailuan Xu, Jinding Chen

**Affiliations:** College of Veterinary Medicine; South China Agricultural University, Guangzhou, People’s Republic of China

## Abstract

Lymphocyte depletion and immunosuppression are typical clinical characteristics of pigs infected with classical swine fever virus (CSFV). The apoptosis of virus-infected and bystander cells plays a role in the immunopathology of classical swine fever (CSF). Here, we offer the first evidence that autophagy is involved in apoptosis and death of T lymphocytes in the spleen of pigs infected with CSFV. Using immunohistochemical assays, we observed that more LC3II-positive cells appear in the T-cell zone of spleens. Spleen cell apoptosis was demonstrated using flow cytometry and TUNEL staining. Confocal immunofluorescence revealed that partial LC3II-positive cells were simultaneously TUNEL-positive. By cultivating spleen cells *ex vivo*, we demonstrated that the inhibition of autophagy by 3-MA treatment inhibited apoptosis and death of T lymphocytes caused by CSFV infection but did not have this effect  on B lymphocytes. Further observations demonstrated that uninfected cells in the spleen were also undergoing autophagy *in vivo*. In summary, these results linked autophagy with the apoptosis and cell death of splenic T cells, providing a new outlook to understand the mechanism of T lymphocyte depletion and immunosuppression during CSF.

## Introduction

Classical swine fever virus (CSFV) is an enveloped RNA virus belonging to the *Pestivirus* genus within the family *Flaviviridae* ^1,2^. CSFV typically causes hemorrhagic syndrome and immunosuppression associated with dengue and hepatitis C virus^3^. Monocytes and macrophages are the main targets for CSFV infection *in vivo* ^4^. However, it is curious that immunosuppression is mainly attributed to the depletion of bystander leukocyte populations, and hemorrhages are not caused by viral replication in endothelial cells^5–7^. These findings complicate the pathological mechanism of CSFV.

Apoptosis, a well-known type I programmed cell death, mediates the elimination of damaged, aberrant or infected cells in multicellular organisms^8^. Leukocyte depletion in the peripheral blood of CSFV-infected pigs is associated with apoptosis. An increase in the number of apoptotic lymphocytes appears as early as 1 day post infection (dpi), which appears before viremia^9^. In addition, apoptosis is also involved in pathological lesions in the thymus, spleen and lymph node of pigs infected by CSFV^10^. Curiously, CSFV does not cause the death of alveolar macrophages and even inhibits the apoptosis of primary porcine endothelial cells in *in vitro* studies^7,11^. These controversial results confuse the cell death mechanism of lymphocytes during CSFV infection and raise the possibility that other cell death mechanisms may be involved.

Autophagy is an evolutionarily conserved degradation process that maintains the metabolic balance and homeostasis of eukaryotes^12,13^. Autophagy-related (ATG) genes are involved in a multistep mechanism to regulate cytoplasmic cargo sequestration inside double-membrane vesicles and delivery to lysosomes for degradation^14^. Although autophagy is described as type II programmed cell death, unlike apoptosis, it occurs independently of the function of caspases in apoptosis pathways^15,16^. By contrast, autophagy can be induced to accomplish cell death when the apoptosis pathway is inhibited^17^. Moreover, common upstream signals can occasionally trigger both autophagy and apoptosis, resulting in cell death^18^. Previously, we demonstrated that CSFV induces autophagy to enhance viral replication and that the autophagy machinery was hijacked to inhibit the apoptosis of host cells^19,20^. However, whether autophagy occurs in host cells of CSFV-infected pigs remains unclear. The relationship between autophagy and cell death pathways during CSFV infection *in vivo* remains unknown.

The spleen, in which CSFV appears earlier and in which large numbers of viral particles reside, contains various types of macrophage and lymphocyte populations that are sorted by biological origin and behavior^21–23^. To uncover the possible mechanism of lymphocyte depletion during CSF, the association between autophagy and apoptosis in spleen cells of pigs infected with CSFV was investigated. The results showed that autophagy and apoptosis pathways were both activated in the spleen of CSFV-infected pigs using western blotting analysis. More LC3II-positive cells appeared in the T-cell zone of spleen paraffin sections. Confocal images revealed that partial LC3II-positive cells were stained by TUNEL. By cultivating spleen cells *ex vivo*, we offered direct evidence that apoptosis and death of T lymphocytes, but not B lymphocytes, can be prevented by the inhibition of autophagy. For the first time, we demonstrate that autophagy is involved in the apoptosis and death of T lymphocytes in the spleen of pigs infected with CSFV. This finding provides a new idea for exploring the mechanism of immunosuppression caused by CSFV infection.

## Results

### Pigs infected with the CSFV Shimen strain exhibited typical clinical symptoms

**Figure 1:**
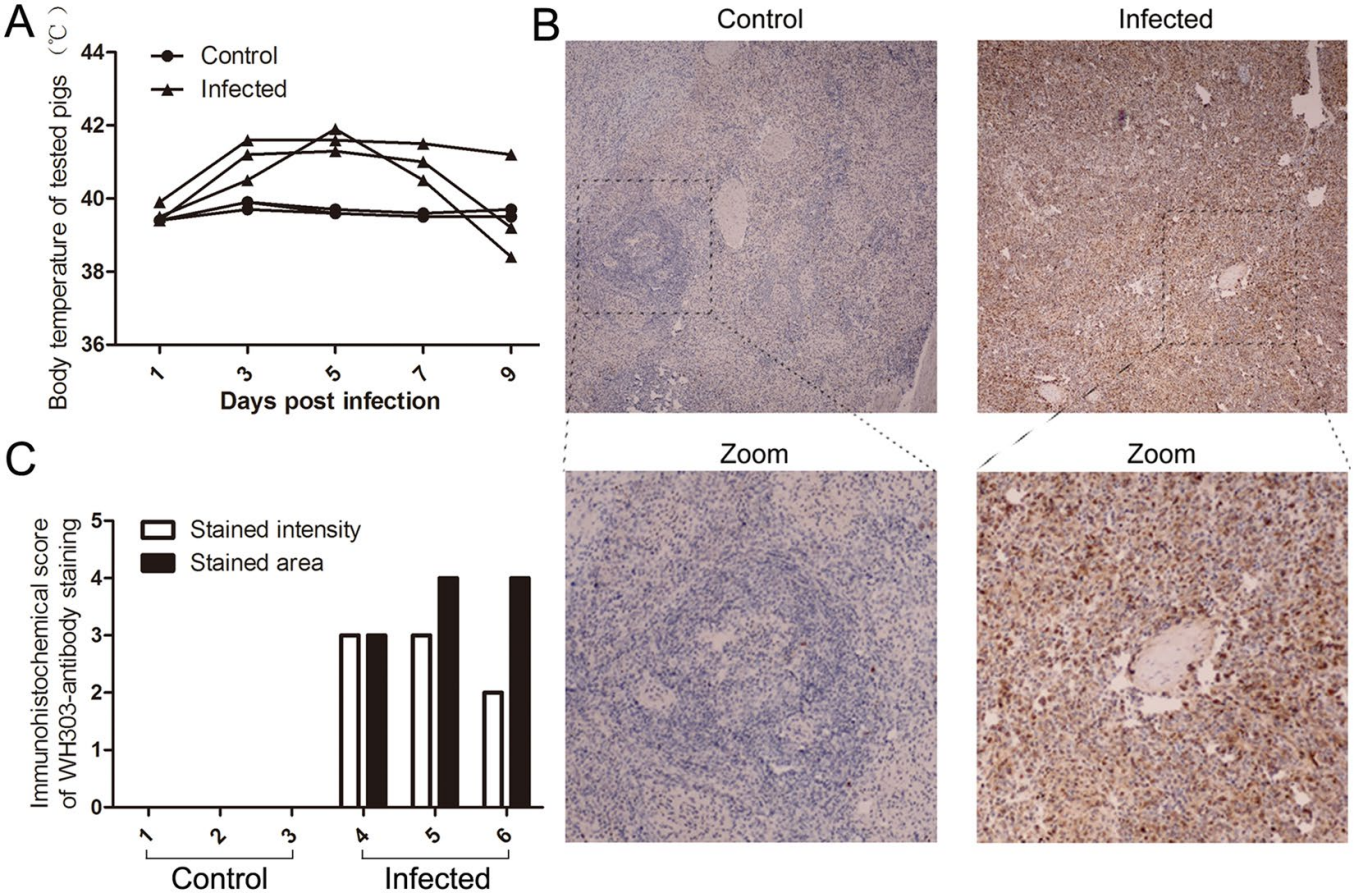
Body temperature of tested pigs and distribution of the CSFV antigen in the spleen. (**A**) Body temperature of tested pigs. 3 pigs were infected, and 3 pigs were used as controls. Each infected pig was intramuscularly injected with 10^5^ TCID_50_ of Shimen strain CSFV. Body temperature was assessed every 2 days until 9 days post infection. **(B)** Location of CSFV in the spleen. Paraffin sections of spleens were stained with mouse monoclonal anti-CSFV E2 protein antibody (1:50) as described in Materials and Methods. The images were a representative immunocytochemistry result captured at magnifications under 20× objectives. **(C)** Immunocytochemistry score. 3 sections of each sample were prepared, and 3 random visual fields of each section were snapped. The score was evaluated by two different clinical doctors. Score of stained intensity: characteristics of positive-stained cells. Pale yellow, tan and sepia were scored as 1, 2, and 3, respectively. Score of stained area: the mean ratios of positive cells in each visual field. Scores of 1, 2, 3, and 4 were corresponded to 6~25%, 26~50%, 51~75% and >75%, respectively.

Pigs inoculated with the CSFV Shimen strain exhibited clinical symptoms of CSF at 3 to 5 dpi in our study. The body temperature of each animal was recorded every two days (Fig. 1A). All animals were sacrificed in accordance with animal ethics guidelines and approved protocols when severe signs appeared at 9 dpi. Typical spleen infarction was observed in CSFV-infected pigs (data not shown). To detect the distribution of CSFV antigen in the spleen, paraffin sections were stained with WH303 antibody, which reacts with E2 protein of CSFV^24^. The immunohistochemical images revealed that most spleen cells exhibited positive staining in infected groups (Fig. 1B and C). By contrast, control groups displayed negative staining (Fig. 1B and C).

### Increased apoptosis of spleen cells in CSFV-infected pigs

**Figure 2:**
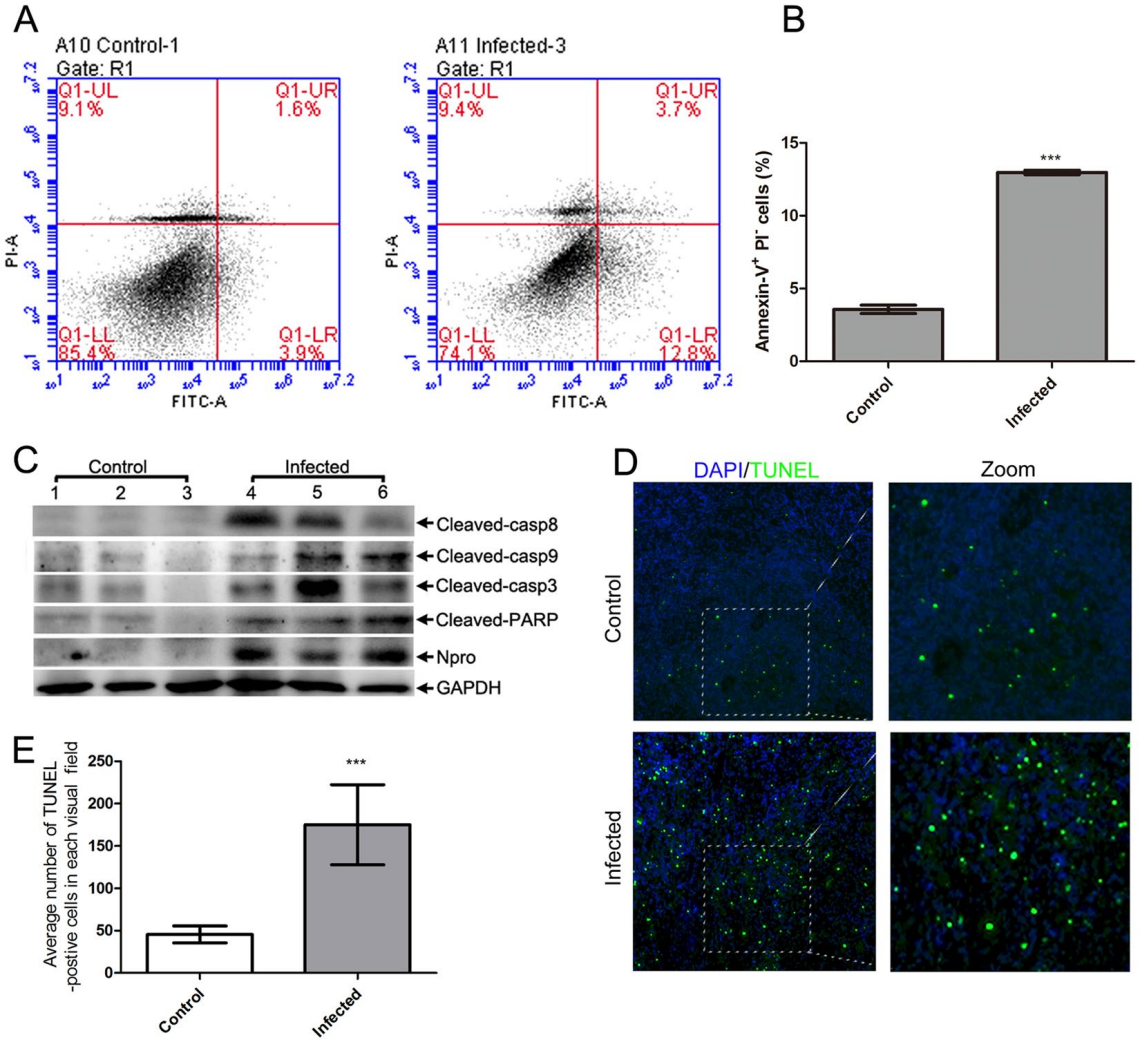
Apoptosis was activated by CSFV infection in the spleen. (**A)** Flow cytometry analysis of cell death in the spleen. Cell death was quantified using FITC-labeled Annexin V and PI. Q1-UL, another dead cell population that was FITC-Annexin V-negative and PI^−^ positive; Q1-UR, a necrotic or end stage apoptotic cell population that was FITC-Annexin V- and PI^−^ positive; Q1-LL, a cell population not undergoing apoptosis that was FITC-Annexin-V- and PI^−^ negative; Q1-LR, an early apoptotic cell population that was FITC-Annexin V-positive and PI^−^ negative. **(B)** Statistical analysis of the ratio of apoptosis (Annexin-V^+^ PI^−^) cells in the spleen (mean ± SD; n = 3; ***p < 0.001). **(C)** Western blotting revealed changes of apoptotic proteins. Cell lysates were prepared as described in Materials and Methods, and antibodies specific for the indicated proteins were used for western blotting analysis. **(D)** TUNEL staining demonstrated the existence of apoptotic cells *in situ*. The paraffin sections were stained with TUNEL as described in Materials and Methods. A representative image captured at magnifications under 20× objective was shown. **(E)** Statistical analysis of the number of TUNEL-positive cells in the spleen. 3 sections of each sample were prepared, and 3 random visual fields of each section were snapped. The number of apoptotic cells in each image was counted using Image-Pro Plus (version 6.0) (mean ± SD; n = 9; ***p < 0.001).

*In vivo*, apoptosis is associated with the immunopathology mechanism of CSF^9,10^. To confirm the involvement of apoptosis in spleen cells of pigs infected  with CSFV, further research was carried out. Given that phagocytic removal of apoptotic cells is rapid^25,26^, the detection of apoptosis *in vivo* is difficult. Therefore, Annexin-V, which binds to phosphatidylserine exposed on the surface of early apoptotic cells, was introduced to evaluate cells that were programmed to die^26^. A representative example of flow cytometry detection of apoptosis in spleen cells is presented in Fig. 2A. Statistical analysis indicated that CSFV obviously increased the frequency of the early apoptotic cell population (Annexin V^+^ PI^−^) (Fig. 2B). To demonstrate that apoptotic signals were activated in spleen cells, hallmark apoptotic proteins were analyzed by immunoblotting. Cleaved caspase-8 and -9 are typically considered as extrinsic and intrinsic initiators, respectively. However, cleaved caspase-3 and PARP are considered as functional downstream effectors^27^. Our results demonstrated that CSFV-mediated up-regulation of cleaved caspase-3 and PARP levels were increased in spleen cells (Figs 2C and S1). In addition, we evaluated caspase-8 and caspase-9 expression to differentiate extrinsic and intrinsic apoptosis. Both initiators were initiated in the spleen cells of pigs infected with CSFV (Figs 2C and S1). To further confirm the presence of apoptotic spleen cells *in situ*, we stained DNA fragmentation of paraffin sections using the terminal deoxynucleotidyl transferase-mediated dUTP nick end labeling (TUNEL) method. An increased ratio of TUNEL-positive cells appeared in the CSFV-infected group when sections were inspected by immunofluorescence microscopy (Fig. 2D and E). Overall, our data demonstrated that CSFV infection caused the apoptosis of cells in the spleen.

### CSFV enhanced the autophagy pathway in spleen cells

**Figure 3:**
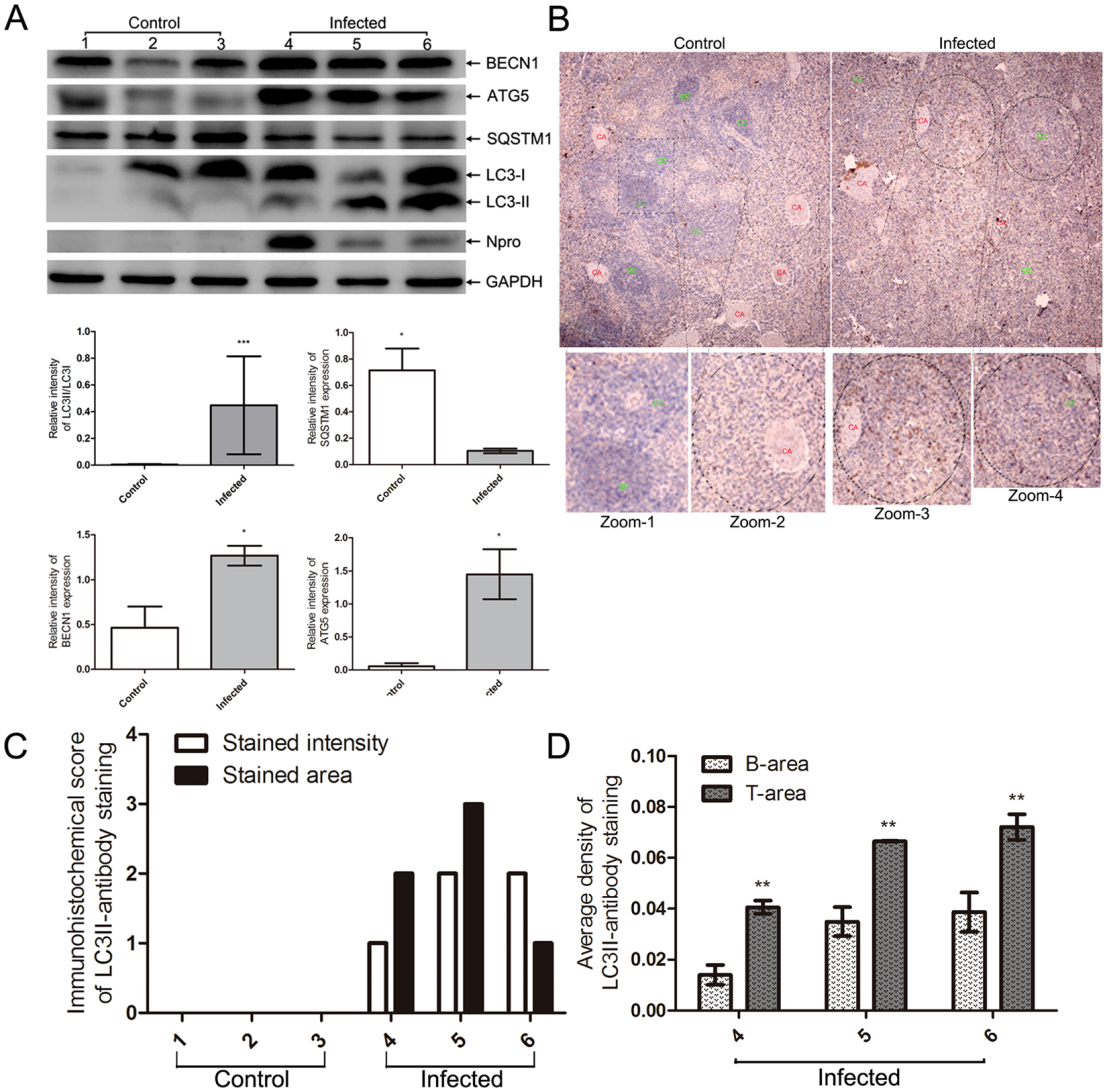
CSFV induced autophagy in splenic cells. (**A)** Analysis of autophagy-related proteins by western blotting. Cell lysates were prepared as described in Materials and Methods, and antibodies specific for the indicated proteins were used for western blotting. The relative levels of target proteins were estimated by densitometry, and the ratios were calculated relative to GAPDH (mean ± SD; n = 3; *p < 0.05, ***p < 0.001). **(B)** Immunochemistry assay revealed the distribution of autophagic cells in the spleen. The paraffin sections were stained with LC3II antibody as described in Materials and Methods. Visual fields of each section were snapped under 20× objectives. **(C)** Immunocytochemistry score. 3 sections of each sample were prepared, and 3 random visual fields of each section were obtained. The score was evaluated by two different clinical doctors. The judging standard was the same as described in Fig. 1C. (**D**) Comparison of the mean optical staining density of T-cell and B-cell zones in the spleen. B-cell and T-cell zones in the spleen were defined according to the structure of the splenic central arteriole and the density of cell populations as described in Materials and Methods. Representative structures of B-cell zone (zoom-1 and zoom-4) and T-cell zone (zoom-2 and zoom-3) were presented in Fig. 3B. The mean optical density of B-cell and T-cell zones was analyzed using Image-Pro Plus (version 6.0) as described in Materials and Methods. The data represent the mean ± SD of 3 random fields of each section (**p < 0.01).

Autophagy is involved in the replication of CSFV *in vitro*, as demonstrated in our previous study^19^. To clarify the effect of autophagy on splenic cells in pigs infected with CSFV, we first evaluated the expression of LC3II and SQSTM1, which were used as markers for assessing autophagy^28^. In contrast to the increased levels of LC3II, which indicate the formation of the autophagosome^29^, SQSTM1 exhibited reduced levels (Fig. 3A), reflecting the increased protein degradation of autolysosomes^30^. Simultaneously, we assessed changes in ATG5, which associates with membranes of precursor autophagosomes^31^. The results revealed that CSFV increased the expression of ATG5 in spleen cells (Fig. 3A). Moreover, increased levels of BECN1, which plays a role in the initiation of autophagy during CSFV infection *in vitro* ^19^, was detected in CSFV-infected spleen cells (Fig. 3A). To strengthen the occurrence of autophagy and to examine the spatial distribution of autophagic cells in the spleen of piglets infected with CSFV, paraffin sections were stained using a LC3II-specific antibody. As presented in Fig. 3B and C, CSFV increased the assessment score of the immunohistochemical assay. Notably, CSFV caused LC3II-positive cells to be mainly distributed around the splenic central arteriole (Fig. 3B, Zoom-2 and Zoom-3), which is a zone of T-lymphocyte predominance (T-cell zone) called the periarteriolar lymphoid sheath (PALS)^32,33^ (Fig. 3B and D). However, relatively few LC3II-positive cells were observed in the area with closely packed monomorphic small lymphocytes surrounding the PALS (Fig. 3B, Zoom-1 and Zoom-4), which is referred to the B-cell zone^32,33^ (Fig. 3B and D). Together, these results demonstrate the association between autophagy and CSFV infection in the spleen. To the best of our knowledge, this is the first data demonstrating that autophagy is activated by CSFV infection *in vivo*.

### Autophagic cells partially underwent apoptosis in the spleen of pigs infected with CSFV

**Figure 4:**
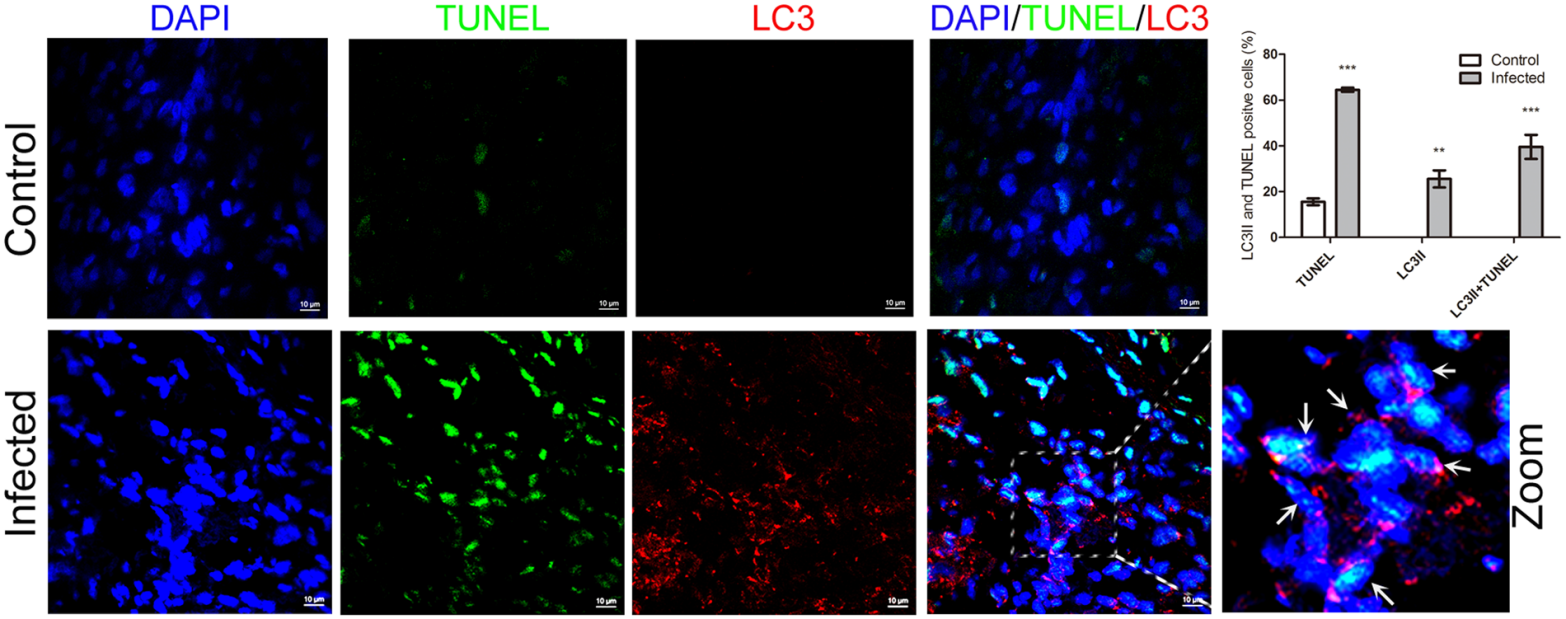
Confocal microscopy showing the connection of autophagic and apoptotic cells. Frozen sections stained with TUNEL (green) were simultaneously immunostained with LC3II-antibody (red). Nuclei was stained with DAPI. In the zoomed images, the arrows indicated the colocalization of cells both undergoing autophagy and apoptosis. The average ratios of TUNEL-positive, LC3II-positive and double stained cells were based on at least 50 cells in each group (mean ± SD; n ≧ 50 cells; **p < 0.01, ***p < 0.001).

Our present data demonstrated that CSFV infection caused autophagy and apoptosis simultaneously in the spleen. Apoptosis is associated with the depletion of lymphocytes^4,9,34^. To clarify the relationship between autophagy and apoptosis of spleen cells in pigs infected with CSFV, frozen spleen sections were double stained with LC3II-antibody and TUNEL. Quantitative analysis of confocal images revealed that more apoptotic cells (~65%) appeared in CSFV-infected spleen compared with autophagic cells (~25%). Notably, partial LC3II-positive cells (~40%) were stained with TUNEL in CSFV-infected spleens (Fig. 4). This finding offered evidence that autophagy may participate in the apoptotic process in spleen cells of CSFV-infected pigs.

### CSFV caused autophagy-mediated apoptosis and cell death in spleen T lymphocytes

**Figure 5:**
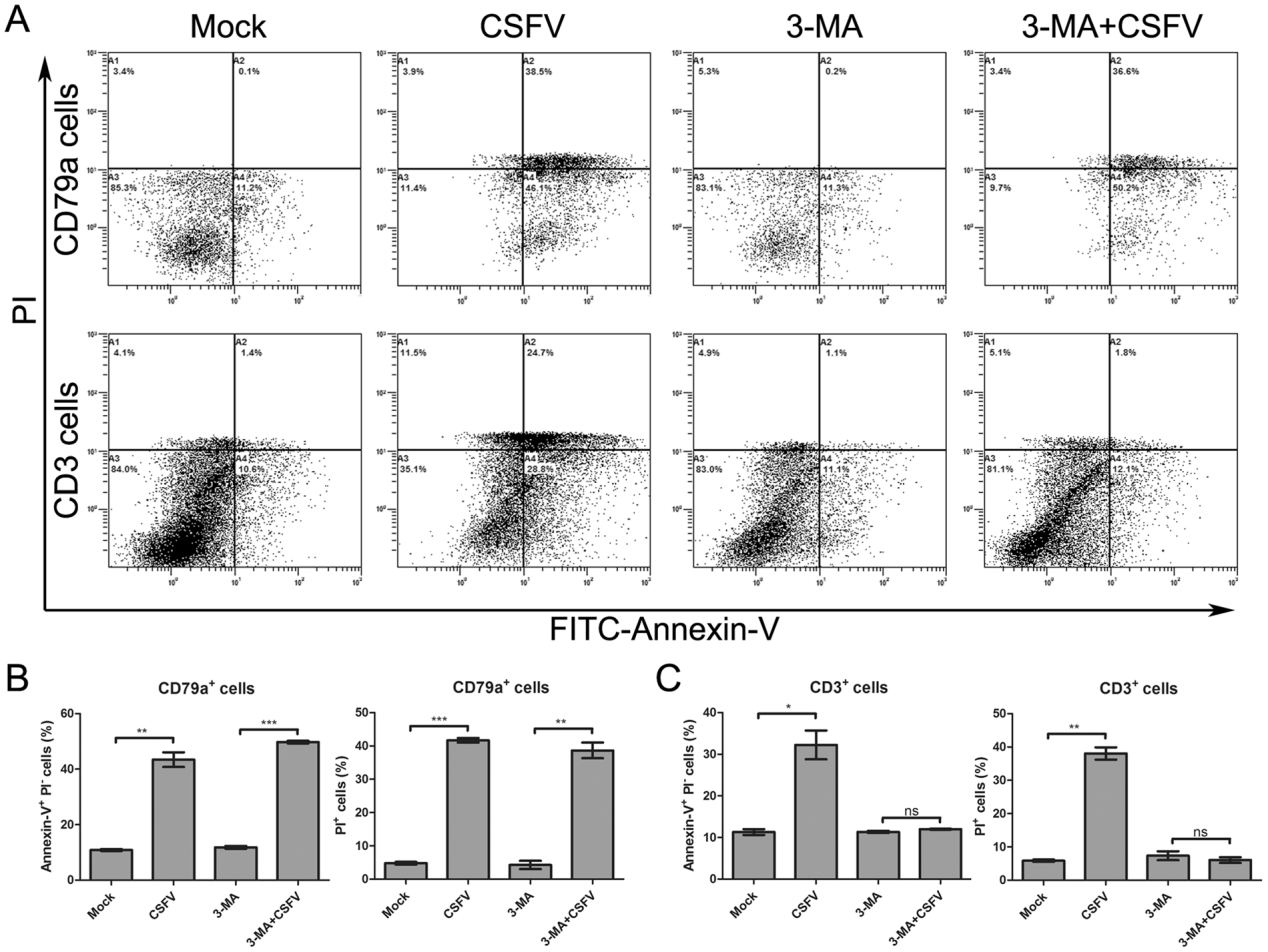
Inhibition of autophagy reduced apoptosis and death of spleen CD3^+^ cells *ex vivo*. (**A**) Flow cytometry analysis of splenic cell death. Spleen cells were prepared and cultivated *ex vivo* as described in Materials and Methods. CSFV infection (MOI = 1) was conducted after cells were pretreated with 3-MA (5 mM) for 4 h. At 3 dpi, cultivated cells were stained with APC-conjugated antibody against CD79a and PE/Cy5-conjugated antibody against CD3 for cell type identification. Then, cells were stained with FITC-Annexin V and PI to analyze cell death. **(B)** Statistical analysis of apoptosis (Annexin-V^+^ PI^−^) and death (PI^+^) ratios of CD79a^+^ cells (mean ± SD; n = 3; **p < 0.01, ***p < 0.001). **(C)** Statistical analysis of apoptosis (Annexin-V^+^ PI^−^) and death (PI^+^) ratios of CD3^+^ cells (mean ± SD; n = 3; *p < 0.05, **p < 0.01).

Based on the findings that LC3II-positive cells were predominant in the T-cell zone of the spleen (Fig. 3B and D) and that partial LC3II-positive cells were also TUNEL-positive in CSFV-infected spleens (Fig. 4), we assessed whether autophagy played a role in the apoptosis of T lymphocytes in the spleen of pigs infected with CSFV. To answer this question, we attempted to culture spleen cells *ex vivo* following the method described previously^35^. The drug 3-methyladenine (3-MA) was used to inhibit autophagy in cultured spleen cells. As shown in Fig. 5, CSFV infection not only increased early apoptosis (Annexin-V^+^) of CD79a^+^ and CD3^+^ cells but also increased cell death (PI^+^). However, the early apoptosis and death of CD3^+^ cells but not CD79a^+^ cells are obviously prevented by 3-MA (Fig. 5B and C). CD79a and CD3 are the special surface receptors of B and T lymphocytes, respectively^36,37^. According to these data, we hypothesized that autophagy resulted in apoptosis and death of T lymphocytes in the spleen of pigs infected with CSFV.

### Autophagy and apoptosis also occurred in bystander spleen cells

**Figure 6:**
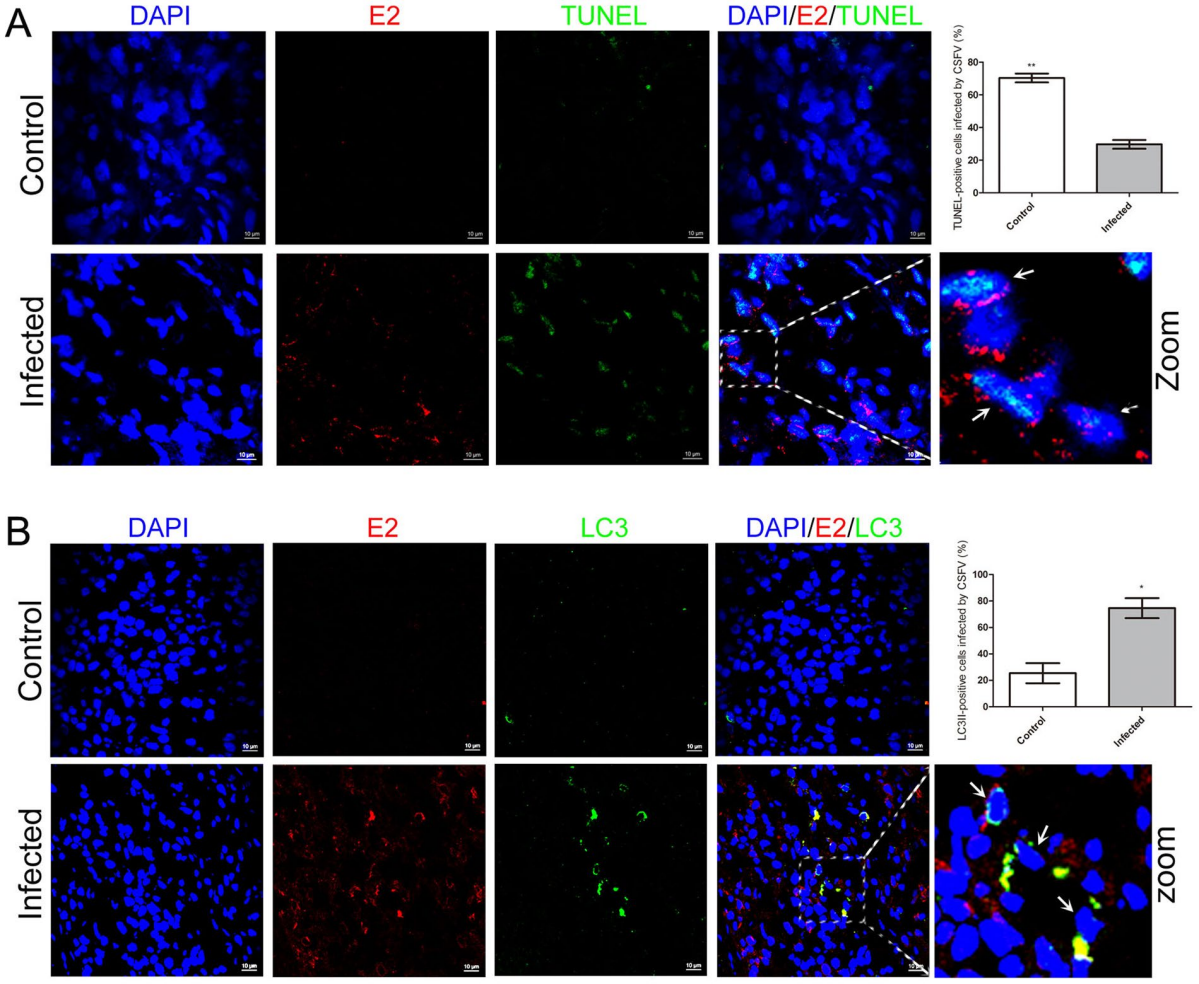
Confocal microscopy showing the colocalization of autophagic cells or apoptotic cells with CSFV. (**A**) Frozen sections were double stained with LC3II-antibody (green) and WH303-antibody (red). Nuclei was stained with DAPI. In the zoomed images, the arrows indicated autophagic cells infected with CSFV. The average ratios of LC3II-positive cells infected with CSFV were based on at least 50 cells in each field (mean ± SD; n ≧ 50 cells; *p < 0.05). **(B)** Frozen sections were double stained with LC3II-antibody (green) and WH303-antibody (red). Nuclei was stained with DAPI. In the zoomed images, the arrows indicated apoptotic cells infected with CSFV. The average ratios of TUNEL-positive cells infected with CSFV were based on at least 50 cells in each field (mean ± SD; n ≧ 50 cells; **p < 0.01).

Previous reports demonstrated that the depleted leukocyte populations are mainly bystanders during CSFV infection *in vivo* ^5,7^, so we further examined whether autophagy and apoptosis in spleen cells were directly caused by CSFV infection. By staining E2 protein with WH303 antibody, confocal immunofluorescence of LC3II or TUNEL staining merged with the CSFV antigen were investigated. Most apoptotic cells were not infected with CSFV *in vivo* (Fig. 6A). However, it is worth noting that a small proportion of autophagic cells were not infected by CSFV, and that not all CSFV-infected cells were undergoing autophagy (Fig. 6B), in contrast to the observation *in vitro* ^19^.

## Discussion

Leukopenia is a typical feature of piglets infected with CSFV, which may ultimately result in immunosuppression^4,38,39^. Although previous reports have linked apoptosis with the depletion of lymphocytes^9,34^, whether other cell death signals play a role in the process remains unknown. As the type II programmed cell death signal, autophagy can exert an independent or cooperative function with apoptosis to determine the fate of cells^40^. Continuous with our previous findings that CSFV induces autophagy to inhibit replication and apoptosis in host cells *in vitro* ^19,20^, we present evidence for the first time that autophagy participates in the apoptosis and death of T lymphocytes in the spleen of pigs infected with CSFV.

In the spleen, apoptosis is associated with pathological lesions caused by CSFV infection^10^. We sought to confirm the apoptosis of splenic cells and to explore the relationship between autophagy and apoptosis in the spleen of CSFV-infected pigs. An increased ratio of Annexin-V positive cells was detected using flow cytometry in the spleen. The results in the previous publication demonstrated that CSFV increased the percentage of cells with low mitochondrial transmembrane potential (ΔΨ*m*) in peripheral blood lymphocytes^9^. Similar to Annexin-V, which binds to phosphatidylserine exposed on the surface of early apoptotic cells^26^, reduction of ΔΨ*m* is also an early marker of apoptotic cells^41^. Previous findings demonstrated that CSFV inhibits apoptosis signals of vascular endothelial cells *in vitro* ^11^. In the present study, extrinsic and intrinsic apoptosis signals were activated using western blotting. The contrasting roles of apoptosis *in vitro* and *in vivo* have long been discussed^9,11^. Furthermore, TUNEL staining *in situ* revealed the frequent emergence of apoptotic cells in the spleen. This finding is consistent with the results demonstrating that TUNEL-positive cells are frequently observed in periarterial lymphatic sheaths (PALS) from 3 dpi^10^.

Previous *in vitro* studies demonstrated that virus-induced autophagy enhances the replication of particles and reduces apoptosis in host cells^42,43^. However, few reports have focused on autophagy and viruses *in vivo*. Whether autophagy is helpful for the existence of the virus in the host is unknown. The changes in autophagy markers (LC3II and SQSTM1) and the increased expression of autophagy-related genes, including ATG5 and BECN1, demonstrated that CSFV enhanced the formation of autophagy bulk and autophagy flux in splenic cells. These findings are consistent with those of a previous *in vitro* study showing that CSFV induced autophagy^19^. Activation of autophagy in CSFV-infected pigs demonstrated that viral immunopathology *in vivo* is related to autophagy or autophagy-related genes^44–46^. Moreover, examination of paraffin sections stained by LC3II-specific antibody demonstrated that autophagic cells were mainly scattered around PALS, which are mainly populated by T lymphocytes and referred to as the T-cell zone based on previous reports^10^. This observation links T cells with autophagy during CSFV infection *in vivo*.

As type II programmed cell death, autophagy is closely related to apoptosis. Autophagy and apoptosis can act as partners, antagonists or enablers to induce cell death in a complex manner^18^. Here, we first observed that autophagic cells were partially undergoing apoptosis simultaneously based on confocal images. This finding suggested that apoptotic cells in the spleen during CSFV infection might be partially caused by autophagy. By cultivating spleen cells *ex vivo*, we demonstrated that inhibition of autophagy by 3-MA treatment prevented apoptosis and death of T lymphocytes, but not B lymphocytes, caused by CSFV infection. Furthermore, we demonstrated that not all cells undergoing autophagy and apoptosis were infected with CSFV. Thus, autophagy and apoptosis signals in bystander cells were activated *in vivo*. These overall results were somewhat consistent with the finding that autophagy results in apoptosis of bystander CD4^+^ T lymphocytes during the development of AIDS^47^. By contrast, CSFV only induced autophagy in infected host cells to enhance viral replication^19^ and to reduce the apoptosis of infected cells *in vitro* ^20^. We hypothesized that the induction of autophagy in bystander cells *in vivo* may be attributed to complex stress inducers of autophagy, including starvation, cytokines, or pathogens^42^. For example, Type I interferons (IFN) are important proteins in the antiviral response, but evidence has highlighted their newly discovered function as inducers of autophagy^48^. Interestingly, up-regulation of IFN-stimulated genes is related to cell death in acute CSFV-infected piglets^49^. However, whether autophagy of bystander cells was induced by high levels of IFN remains to be clarified.

In conclusion, the present study offered the first data that apoptosis and death of T lymphocytes in the spleen are dependent on the activation of autophagy in CSFV-infected pigs. We explored a new approach for understanding the depletion of the T cell population during CSFV infection *in vivo*, which is associated with immunosuppression in pigs. Our findings may be helpful to clarify the pathogenesis of CSFV infection and to develop novel antiviral strategies. Chemical drugs target to ATG proteins may be potential modulators for treatment of T leukocytes depletion of pigs caused by CSFV infection. However, future studies should focus on how autophagy is involved in apoptosis and death of T cells and why B cells do not undergo autophagy in this context.

## Materials and Methods

### Antibodies

The following primary antibodies were used in the study: rabbit polyclonal anti-LC3B (Cell Signaling Technology, 2775), rabbit polyclonal anti-SQSTM1/p62 (Sigma, SAB2104334), rabbit polyclonal anti-BECN1 (ABclonal, A7353), rabbit polyclonal anti-ATG5 (ABclonal, A0203), mouse monoclonal anti-GAPDH (Beyotime, AG019), rabbit polyclonal anti-caspase-8 (Beyotime, AC056), rabbit polyclonal anti-caspase-9 (Beyotime, AC062), rabbit polyclonal anti-Caspase-3 (ABclonal, A2156), rabbit polyclonal anti-PARP (Beyotime, AP102) and mouse monoclonal anti-CSFV E2 (WH303) (JBT, 9011). Mouse polyclonal anti-CSFV Npro was kindly provided by Dr. Xinglong Yu (Veterinary Department, Hunan Agricultural University, China). The secondary antibodies used for immunoblotting analysis were HRP-conjugated goat anti-mouse IgG (Bioworld, BS12478), HRP-conjugated goat anti-rabbit IgG (Bioworld, BS13278). The secondary antibodies used for immunofluorescence including Dylight 488 goat anti-mouse IgG (EarthOx, E032210), Dylight 488 goat anti-rabbit IgG (EarthOx, E032220), Dylight 549 goat anti-mouse IgG (EarthOx, E032310) and Dylight 549 goat anti-rabbit IgG (EarthOx, E032320).

### Virus

Blood stock of the virulent CSFV strain Shimen was propagated 1 passage in the swine kidney cell line PK-15. Virus titers were determined by end-point titration on PK-15 cells as previously described^19^. Briefly, cells cultivated in 96-well plates were fixed with 80% acetone at −20 °C for 30 min, and infected cells were detected using the immunofluorescence assay. The primary antibody was mouse anti-CSFV E2 antibody (WH303), and the secondary antibody was Dylight488-conjugated goat anti-mouse IgG. Virus titers were calculated according to Kaerber and expressed as 50% tissue culture infectious doses (TCID_50_) per milliliter.

### Infection of pigs

A total of 6 animals (Tibet swine, 5 months old) bred at the specific pathogen-free (SPF) unit of the institute were assessed using ELISA and PCR (data not shown). 3 animals were randomly selected, and each one was infected with 10^5^ TCID_50_ CSFV. The other 3 animals were intramuscularly injected with the same dose of saline water and used as controls. Examination of body temperature and clinical symptoms were conducted every 2 days. All animals were sacrificed at 9 dpi for the collection of spleens when severe clinical symptoms appeared in most infected animals. All experimental procedures were performed in accordance with animal ethics guidelines and approved by the Animal Care and Use Committee of South China Agriculture University, China. The issue number is 2014-12.

### Culture and infection of spleen cells *ex vivo*

Spleen cells were prepared and cultivated following the methods previously described^35^. Briefly, the spleens of anti-CSFV antibody-negative pigs were removed in sterile conditions. The spleen tissue was cut, softly pressed and dispersed by passing through 70-μm cell strainers. Dispersed cells were washed twice in RPMI 1640 supplemented with 2% antibiotics. Approximately 2 × 10^6^ cells were cultivated in a volume of 200 μl culture medium in 96-well U-bottom plates. The culture medium contained RPMI 1640 supplemented with 15% FBS (Gibco) and 1% antibiotics (Gibco). After 18 h, cells were pretreated with 5 mM 3-MA for 4 h followed by inoculating with CSFV at 1 multiplicity of infection for 1 h. Uninfected virus was removed by washing cells twice in RPMI 1640 supplemented with 2% antibiotics. Then, cells continued cultivating in complete medium containing 5 mM 3-MA for 3 days at 37 °C with 5% CO_2_.

### Immunoblotting

After washing spleens samples with PBS three times, 50 to 60 mg small mass of each sample was incubated on ice with 1 ml RIPA lysis buffer (Beyotime, P0013B) containing 1 mM PMSF (Beyotime, ST506) and homogenized by *Dounce* Tissue Grinders. The precipitation was removed by centrifugation at 14,000 g for 10 min at 4 °C. Then, the supernatant was collected, and the protein concentration was quantified by the BCA protein assay kit (Thermo, 23227). Equal amounts of total proteins (20 μg) were separated by 12% SDS-PAGE gels and then electrotransferred onto polyvinylidene fluoride (PVDF) membranes (Millipore, IPVH00010). Then, 5% nonfat milk dissolved in PBS containing 0.1% Tween 20 was used to block the membranes for 1 h at 25 °C. Membranes were incubated with the corresponding primary antibodies at 4 °C overnight and secondary antibodies conjugated to HRP at 37 °C for 1 h. The bands were detected using the ECL Plus kit (Beyotime, P0018) and visualized using the chemiluminescence imaging system (Fine-do × 6, Tanon). Protein blots were measured with Image-Pro Plus (version 6.0).

### Immunohistochemistry

Samples of spleen were fixed in 10% phosphate-buffered formalin, embedded in paraffin, and sliced at 3 to 4 mm. Tissue sections were deparaffinized by 3 steps in xylol at 5 min per step; two steps of 5 min each in absolute ethanol; and one 4-min step in 95% ethanol, 85% ethanol and 70% ethanol each followed by 2 min in distilled water. Antigen retrieval with pH 8.0 EDTA buffer was conducted in the pressure cooker (5 min from the beginning of boiling). Afterwards, samples were incubated with 3% hydrogen peroxide for 8 min to block endogenous peroxidase activity. To examine autophagy activation, paraffin sections were stained with rabbit polyclonal anti-LC3II antibody (1:400). For the detection of cells infected with CSFV, mouse monoclonal anti-CSFV E2 protein antibody (1:50) was utilized. ChemMate EnVision kit (DAKO) was used to display the staining. After counterstaining with hematoxylin, 3 digital images for each section at 3456 × 2304-pixel resolution at magnifications under 20× objectives were captured (Olympus, BX43).

### Evaluation of immunohistochemistry images

The score of immunohistochemistry images was judged by two different clinical doctors according to the criteria reported previously^50^. The staining pattern is not given a serially numerical score but is only assigned a broad category. The score of staining intensity is judged according to characteristics of positively stained cells. Pale yellow, tan and sepia were given scores of 1, 2 and 3, respectively. The score of the stained area was judged according to the ratio of positive cells. Scores of 1, 2, 3, and 4 corresponded to ratios of 6~25%, 26~50%, 51~75% and >75%, respectively.

Semi-quantitative evaluation of IHC staining by Image-Pro Plus (version 6.0) was conducted according to the method introduced in a previous report^51^. Briefly, after B-cell and T-cell zones were defined according to the structure of the splenic central arteriole and the packed density of the cell populations^32,33^, parameters, including area sum and IOD, were measured. The optical density of the area of interest was calibrated and established as follows: hue, 0 to 30; saturation, 0 to 255; intensity, 0 to 255. The mean optical staining density of B-cell and T-cell zones was calculated by IOD/area sum.

### TUNEL staining

To analyze the distribution of cells undergoing apoptosis, paraffin sections of spleen were stained with TUNEL BrightGreen Apoptosis Detection Kit (Vazyme, A112-01/02/03) according to the manufacturer’s instructions. 3 digital images for each section at 3456 × 2304-pixel resolution at magnifications under 20× objectives were captured (Olympus, BX53).

### FACS Analysis

For spleen tissues, partial spleen blocks were minced and passed through a 70-μm cell strainer. Dispersed tissues were washed in phosphate-buffered saline 3 times and then directly stained with FITC-conjugated Annexin-V and propidium iodide (PI) for 10 min to analyze apoptosis using the Annexin V-FITC Apoptosis Detection Kit I (Vazyme, A211-01/02) according to the manufacturer’s instructions.

For spleen cells cultivated *ex vivo*, cells washed in phosphate-buffered saline 3 times were respectively stained with APC-conjugated antibody against CD79a (Abnova, MAB4515) and PE/Cy5-conjugated antibody against CD3 (Abcam, ab25535) at 37 °C for 1 hour. After cells were washed in phosphate-buffered saline 3 times again, the Annexin V-FITC Apoptosis Detection Kit I was used to analyze apoptosis as described above.

### Confocal microscopy

Spleen samples were sectioned (8 μm) on a cryostat. After fixation in 4% paraformaldehyde and permeabilization with 0.25% TritonX-100, cryostat sections were double-stained for the immunofluorescence assay. In brief, mouse monoclonal anti-CSFV E2 protein (1:50) and anti-mouse secondary antibodies conjugated to Dylight-549 (1:200) were used to determine CSFV infection. Anti-LC3B antibody (1:400) and then anti-rabbit secondary antibodies conjugated to Dylight-488 (1:200) or Dylight-549 (1:200) were stained to detect the formation of autophagy. To stain apoptotic cells, TUNEL BrightGreen Apoptosis Detection Kit (Vazyme, A112-01/02/03) was used according to the manufacturer’s instructions. Wherever indicated, nuclei were stained with DAPI (Beyotime, C1005). The fluorescence signals were visualized under a 100× oil objective using LSM710 confocal microscope (Leica).

### Statistical analysis

Statistical analysis was performed utilizing Graph Pad Prism 5 software. Unpaired Student’s t-test was used to compare means of different groups. Value of P lesser than 0.05 were considered significant and that lesser than 0.001 were considered highly significant.

## Electronic supplementary material

Supplemental Figure 1
