# Supplementary material for: Autophagy induces apoptosis and death of T lymphocytes in the spleen of pigs infected with CSFV
Source: Sci Rep. 2017 Oct 19;7:13577. doi: 10.1038/s41598-017-14082-9 (PMC5648758; doi:10.1038/s41598-017-14082-9)
Supplement: Supplementary file 1 — Supplemental Figure 1 [file 41598_2017_14082_MOESM1_ESM.doc]

Autophagy induces apoptosis and death of T lymphocytes in the spleen of pigs infected with CSFV

Hongchao Gou^#^, Mingqiu Zhao^#^, Shuangqi Fan, Jin Yuan, Jiedan Liao, Wencheng He, Hailuan Xu, Jinding Chen*

College of Veterinary Medicine; South China Agricultural University; Guangzhou, People’s Republic of China

^#^These authors contributed equally to this work.

*Correspondence to: Jinding Chen; College of Veterinary Medicine; South China Agricultural University; No.483, Wushan Road; Tianhe District; Guangzhou 510642, People’s Republic of China;

Tel.: +86 20 85288017; Fax: +86 20 85280245; Email: jdchen@scau.edu.cn


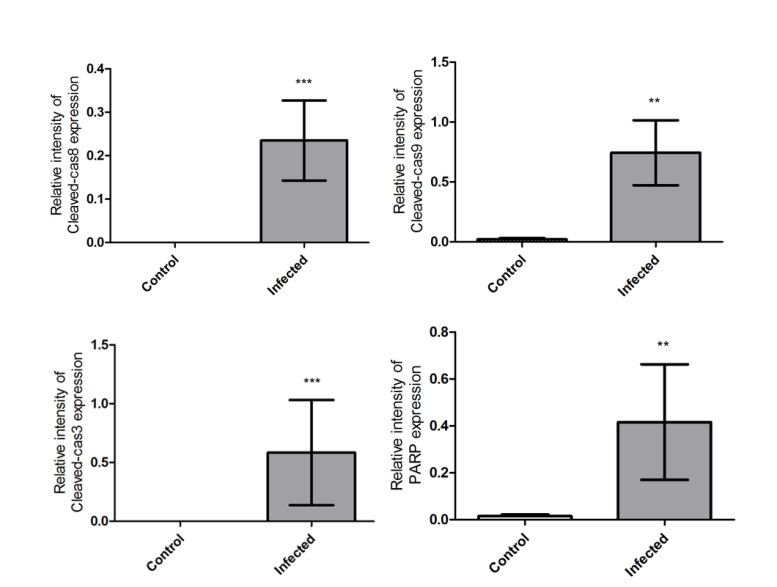


**Supplemental Figure 1. Expression of apoptosis-related proteins in spleen cells of pigs infected with CSFV.** The relative levels of the apoptotic proteins were estimated by densitometry, and the ratios were calculated relative to GAPDH (mean±SD; n = 3; **p＜0.01, ***p＜0.001).
